# Disentangling Temporal and Environmental Effects on Hematological Values in Prairie Rattlesnakes (*Crotalus viridis*)

**DOI:** 10.1002/jez.70105

**Published:** 2026-05-28

**Authors:** Ethan J. Kessler, Sarah J. Baker, Ellen Haynes, Amy N. Schnelle, Matthew C. Allender

**Affiliations:** ^1^ Illinois Natural History Survey, Prairie Research Institute University of Illinois Champaign Illinois USA; ^2^ Wildlife Epidemiology Laboratory, College of Veterinary Medicine University of Illinois Urbana Illinois USA; ^3^ Veterinary Diagnostic Laboratory, College of Veterinary Medicine University of Illinois Urbana Illinois USA; ^4^ Brookfield Zoo Chicago Brookfield Illinois USA

**Keywords:** *Crotalus viridis*, ectotherm, hematology, prairie rattlesnake, seasonality, white blood cells

## Abstract

Hematologic assessment is a common clinical tool used to characterize both individual and population health. In ectothermic organisms, the hematologic response is influenced by external factors such as temperature and season, especially in temperate species. Yet, specific environmental effects have received little direct attention. Therefore, to improve the diagnostic utility of hematologic assessments in reptiles, it is imperative to characterize physiological variation in diagnostic parameters. Our objective was to determine the impacts of temperature and season on commonly assessed components of the complete blood count, including packed cell volume (PCV), total solids (TS) and white blood cell count (WBC). We used a group of 17 long‐term captive Prairie Rattlesnakes (*Crotalus viridis*). Animals were randomly assigned to either a control group kept in an environmental chamber at a constant temperature of 25°C or an experimental group housed in an environmental chamber which reflected average ambient temperature throughout the year (5°C–32°C). We collected blood samples twice monthly for 13 months. We found WBC decreased as the mean previous 14‐day temperature increased, with no effect of season. Changes in total WBC were driven by increased numbers of lymphocytes and decreased number of heterophils and azurophils at higher temperatures. We found a difference in the effect of time since the start of the experiment between control and experimental groups in PCV, with no change in our control group, but a decrease in our experimental group through time. Total solids were higher in the control group, but there was no direct effect of temperature or season. Therefore, we conclude environmental conditions, rather than season, drive variation in hematologic parameters of otherwise healthy snakes and should be included in the interpretation of hematologic assessments in wild reptiles.

## Summary

Temperature‐associated environmental conditions, rather than temporal cycles, drive hematologic variation in prairie rattlesnakes. Temperature‐specific reference ranges may be needed for accurate clinical interpretation in reptiles.

## 1 Introduction

Evaluating wildlife health has become an increasingly important component of conservation actions, given the threat that emerging infectious diseases pose to biodiversity (Lafferty and Gerber 2002). Conservation planning largely focuses on habitat management, predator control, and maintenance of genetic diversity, with wildlife health evaluations often absent or lacking. Furthermore, wildlife disease management activities are almost exclusively reactive, by responding once an outbreak has occurred, rather than proactive health surveillance. Infectious and non‐infectious diseases are often correlated with or compounded by other threats, exacerbating conservation challenges (Lafferty and Gerber 2002). Therefore, incorporating methods to evaluate the overall health of a population or individuals can be informative for conservation programs and lead to projects that monitor wellness rather than documenting disease.

Reptile populations are declining worldwide (Gibbons et al. 2000), and species are vulnerable to infection from a variety of pathogens, including ranavirus, herpesvirus, *Mycoplasma*, and *Ophidiomyces ophidiicola* (Allender et al. 2011; Sim et al. 2016). Due to the increasing regularity of reptile‐focused conservation and management activities in response to biodiversity threats, there is a need to increase health and disease monitoring. Hematologic assessments, modeled after those for endothermic animals, are one of the most common clinical tools for evaluating individual and population health in reptiles, yet diagnostic interpretation remains challenging because multiple intrinsic and extrinsic factors influence blood parameters (Stacy et al. 2011; Sykes and Klaphake 2015; Giori et al. 2020). A single complete blood count result in a reptile is rarely helpful in identifying disease, but serial samples or comparisons among similarly grouped animals may detect significant changes. Several studies have reported reference ranges for free‐ranging reptiles (Alleman et al. 1992; Andreani et al. 2014; Way Rose and Allender 2011; Gottender and Jacobson 1995; Yang et al. 2014; Dickinson et al. 2002; Chung et al. 2009; Hidalgo‐Vila et al. 2007), with others demonstrating the effect of season, age class, sex, venipuncture site, and disease status (Sykes and Klaphake 2015; Stacy et al. 2011; Norton and Allender 2020). Further, as physiological processes in ectotherms are influenced by temperature, it is likely that season and/or temperature at sample collection would affect individual CBC results, thus diminishing the comparability of results to established baselines or between individuals across time (Stacy et al. 2011). Finally, many reptiles undergo periods of seasonal dormancy either due to extreme temperatures or during dry seasons. Dormancy includes periods of fasting and lowered metabolic rate, which may also affect the interpretation of CBC results (Campbell 2015).

Hematological parameters in reptiles are influenced by a suite of intrinsic factors, including species, sex, age, and reproductive status, as well as extrinsic factors such as temperature, photoperiod, diet, and captive versus free‐ranging conditions (Campbell 2005; Stacy et al. 2011; Giori et al. 2020). Seasonal variation in blood parameters has been documented across diverse reptilian taxa. In snakes, seasonal changes in red blood cell counts, hemoglobin, and hematocrit have been linked to breeding cycles, hibernation, and fluctuating environmental conditions (Wojtaszek 1992; Troiano et al. 1997). Similar seasonal patterns in plasma biochemistry have been reported in lizards, with sex‐dependent variations in globulins, cholesterol, and calcium reflecting reproductive activity (Tamukai et al. 2011). Erythrocyte indicators of health status in vertebrates are broadly recognized as useful but complex metrics, as both high and low values can be associated with different stressors, making interpretation context‐dependent (Johnstone et al. 2017). These complexities highlight the need for controlled experimental studies that can isolate environmental drivers of hematologic variation.

Beyond direct temperature effects, environmental conditions that covary wth temperature in temperate systems, such as feeding schedules and photoperiod, may independently influence hematological parameters. Food deprivation in snakes elevates plasma corticosterone, decreases hematocrit and circulating triglycerides, and alters protein catabolism markers, even over relatively short periods (Webb et al. 2017). In chelonians, innate immune function varies with temperature and season in species‐specific ways, and bactericidal ability against certain pathogens appears sensitive to the thermal environment experienced over weekly to monthly timescales (Hartzheim et al. 2023). These findings underscore the importance of considering nutritional status and thermal history when interpreting hematological data from ectotherms, as periods of reduced temperature often coincide with fasting and shifts in photoperiod.

While previous studies have documented hematologic changes in response to seasonal variation in hormone circulation (Muñoz et al. 2000), most studies describing seasonal variation in hematologic parameters typically report on free‐ranging individuals and thus use “season” as a proxy for temperature where “winter” samples are from the coldest months and “summer” samples are from the warmest (Campbell 2005; Nardini et al. 2013). However, it is unclear whether the variance observed in seasonal studies is due to proximate temperature‐related responses or to a temporal seasonal cycle. Furthermore, in temperate species, seasonal environmental changes encompass not only temperature, but also photoperiod and associated shifts in feeding behavior, metabolic rate, and reproductive physiology (Wojtaszek 1992). These factors are typically confounded in field studies, making it difficult to determine which environmental variables are most important for hematologic variation.

Our objective was to separate intrinsic temporal variation from the suite of environmental changes associated with seasonal temperature cycles to determine which is most important for determining hematologic values. Using an experimental approach with a common and widely distributed temperate pit‐viper, the Prairie Rattlesnake (*Crotalus viridis*), we maintained a control group at constant temperature and photoperiod with continuous feeding for comparison with an experimental group experiencing seasonally cycling temperature, photoperiod, and feeding regimes. This design allowed us to compare a group experiencing seasonally associated environmental change against a group exposed only to the temporal aspect of seasonal change. Although this study was conducted in a single species under one set of captive conditions, the study provides a foundation for understanding how environmental drivers shape hematologic variation in temperate reptiles.

## 2 Materials and Methods

We used 17 adult, long‐term captive Prairie Rattlesnakes (10 males, 7 females) randomly assigned to control or experimental groups (ten snakes in the experimental group and seven in the control group). Males and females were distributed across groups as follows: six males and four females in the experimental group and four males and three females in the control group. All snakes had been maintained in captivity for a minimum of 5 years prior to the start of the study. We held the control group at a constant temperature of 25°C and varied the temperature of the experimental group weekly from 5°C to 32°C according to the average weekly temperature for Champaign, IL, USA (the location of the study). Temperature changes for the experimental group were made gradually over a 12‐h period at the start of each week and temperatures were monitored continuously using thermometers housed within the chambers.

The control group was held at a constant 12 L:12D photoperiod throughout the 12‐month project period. Photoperiod for the experimental group was adjusted weekly to match ambient daylight hours for Champaign, IL using automated timers to ensure snakes were exposed to average weekly photoperiod duration during each week. All snakes were housed in plastic Neodesha cages (Neodesha Plastics Inc., Neodesha, KS, USA) on newspaper substrate with a hide box and access to water ad libitum. We fed the snakes pre‐killed mice every 2 weeks. Feeding the experimental group ceased when temperatures were below 20°C. This feeding protocol ensured that snakes were not fed meals when ambient temperatures prevented adequate digestion to prevent digestive failure and septicemia.

We collected blood samples from all snakes twice per month. Snakes were removed from their enclosures and restrained using tubes for venipuncture. Within 5 min of removal from the enclosure, we collected 0.2 mL of blood from the caudal vein using a 22 ga needle and transferred it immediately to a lithium heparin microtainer (Becton Dickinson, Franklin Groves, NJ). Samples with noticeable lymph‐contamination, hemolysis, or clots were discarded, and a new sample was collected. We placed all samples on ice after collection and conducted complete blood counts (CBCs) within 4 h of sample collection.

We used the avian leukopet system (VetLab Supply, Palmetto Bay, FL, USA) to determine white blood cell counts (WBC) on a Bright‐line hemacytometer (Hausser Scientific, Horsham, PA, USA). This indirect method uses phloxine B dye to differentially stain eosinophils and heterophils in whole blood and has been widely used and validated for reptilian leukocyte estimation (Campbell 2015; Stacy et al. 2011; Sykes and Klaphake 2015). We then performed a 100‐cell differential WBC from a blood smear slide preparation stained with Hema 3 (Fisher Scientific, Pittsburgh, PA, USA). Differential counts were determined by a single observer (ANS). Absolute counts for each leukocyte type were then calculated using a standard equation for the avian leukopet system results in conjunction with the white blood cell differential counts (Reilly et al. 2024).

We performed packed cell volume (PCV) and total solids (TS) analysis by filling two microhematocrit tubes (Thermo Fisher Scientific Inc., Waltham, MA, USA) with whole blood. We centrifuged each microhematocrit sample (6000 rpm x 5 min) and recorded PCV (% volume of red blood cells). We determined TS using a hand‐held analog refractometer (Amscope RHC‐200ATC refractometer, National Industry Supply, Torrance, CA, USA) using plasma from microhematocrit tubes after centrifugation.

### 2.1 Statistical Analysis

We used mixed effects general linear models to determine whether season, current temperature, or mean temperature 3, 7, or 14 days prior to sampling best explained hematological parameters. We selected these three temporal windows (3, 7, and 14 days) to evaluate whether hematological responses were driven by acute thermal conditions near the time of sampling or across a longer preceding period. We also tested days since the first blood collection to identify any cumulative effects of biweekly blood draws.

We created single‐parameter models of each explanatory variable, including log‐transformed PCV, WBC, and TS. Season was parameterized as a day of year transformed as a circular variable to mimic the cyclical nature of season. Snake ID was included as a random effect to control for individual variation in repeated samples. Candidate models were compared using Akaike's Information Criterion corrected for small sample sizes (AIC_c_), and models with the lowest AIC_c_ score were identified as the top model (Burnham and Anderson 2002). Due to the collinear nature of our temperature variables, only the top temperature‐based model was considered when interpreting ΔAIC_c_ values.

We used a redundancy analysis (RDA) to investigate which white blood cells drive variation in total WBC. RDA is an asymmetrical multivariate technique analogous to linear regression that models variation in a matrix of dependent variables (e.g., differential WBC counts) by a matrix of independent variables (Legendre and Legendre 2012). RDA is commonly used in ecology to determine which environmental or spatial variables (independent variables) influence the community at a sampling location (dependent variables). Differential white blood cell counts were chi‐square transformed (Legendre and Gallagher 2001) and modeled by the best‐fit temperature variable and season. Season was modeled as the *sin* and *cos* of the day of the year (converted to radians) to preserve the circular nature of this variable. In this situation, *sin* of day of year is highest in spring and lowest in fall, while *cos* of day of year is highest in winter and lowest in summer. Therefore, *sin* and *cos* of the day of year will hereafter be referred to as Spring and Winter, respectively. The significance of the entire RDA model and individual axes was determined via permutation tests. The significance of independent variables was determined via marginal testing.

All analyses were undertaken in R v. 4.4.2 (R Core Team 2024). The package *lme4* (Bates et al. 2015) was used for mixed effects models, and the *vegan* package (Oksanen et al. 2019) was used for all multivariate analyses.

## 3 Results

**Figure 1:**
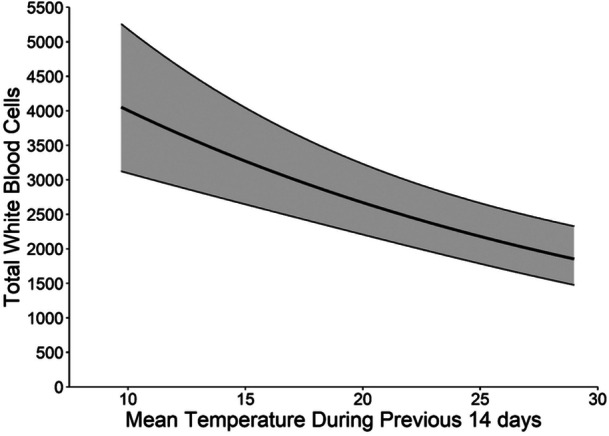
Modeled estimates and 95% confidence intervals of *C. viridis* total white blood cell counts (WBC) by mean temperature in the 14 days preceding blood sample. The solid black midline represents modeled estimates and 95% confidence intervals are in gray.

**Figure 2:**
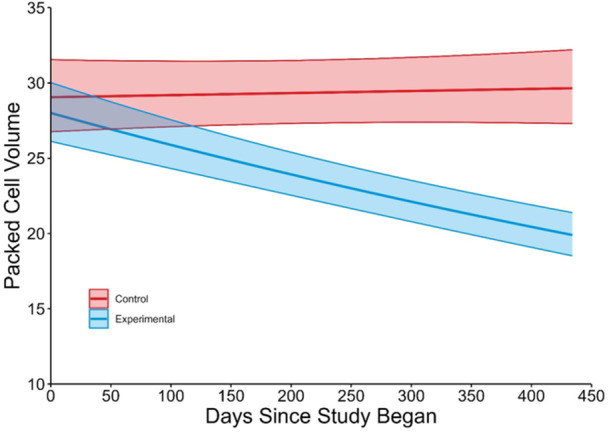
Modeled estimates and 95% confidence intervals of *C. viridis* packed cell volume (PCV) by days since the study began for the control (red) and experimental groups (blue). The solid midlines represent modeled estimates and shaded areas represent 95% confidence intervals.

**Figure 3:**
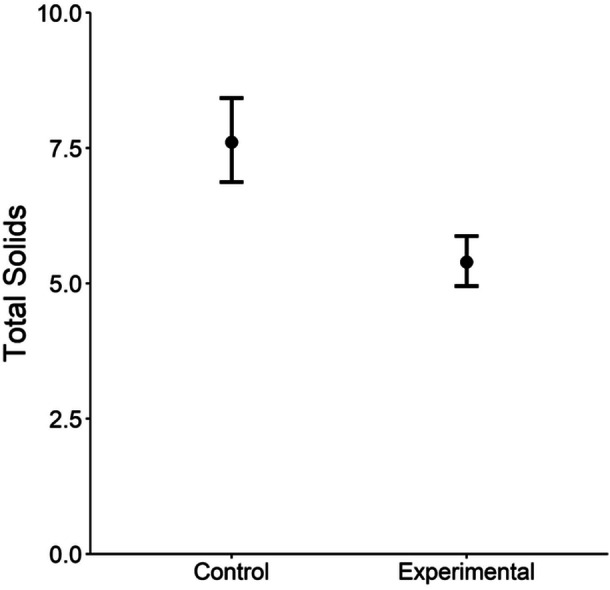
Modeled estimates and 95% confidence intervals of *C. viridis* total solids (TS) by group (Control vs. Experimental). Points represent modeled estimates while error bars represent 95% confidence intervals.

We collected 374 blood samples across the 17 snakes (150 control samples and 224 experimental samples), resulting in a median of 22 samples per snake (range = 18–25). Variation in sample number collected from each individual was due to clotting of samples and occasional inability to obtain blood. Temperature best explained WBC, with mean temperature 14‐days prior to sampling as the top model in each (Table 1 a). As temperature increased, WBC decreased (Figure 1). From the initial model set, PCV was best explained by the number of days since first blood collection; however, further evaluation of the data showed a potential interactive effect of treatment group (control or experimental) on days since first blood collection, therefore this *post hoc* model was added to the model set and showed the best fit (Table 1 b). The interaction between treatment group and days since first blood collection had steady PCV values throughout the study for the control group, but the experimental group had a gradual decrease in PCV over time (Figure 2). Treatment group (control or experimental) was the top model explaining variation in TS (Table 1 c) and the control group had significantly higher TS than the experimental group (Figure 3).

**Table 1:** Candidate models explaining variation in (a) total white blood cell counts (WBC), (b) packed cell volume (PCV), and (c) total solids (TS) in captive *Crotalus viridis*. Models consist of mean temperatures in previous 3 (Temp.prev.3), 7 (Temp.prev.7), and 14 days (Temp.prev.14), temperature day of sampling (Temp), seasonal effect modeled via circular transformation of day of year (Season), group (control vs. experimental; Group), and intercept‐only (Intercept).

(a)					
WBC model	AIC_c_	ΔAIC_c_	Cum. *w*AIC_c_	Marginal *R* ^2^	Conditional *R* ^2^
Temp.prev.14	861.46	0.00	0.40	0.06	0.25
Temp.prev.7	862.31	0.85	0.67	0.06	0.25
Temp.prev.3	863.37	1.92	0.82	0.06	0.25
Temp	863.56	2.10	0.97	0.06	0.25
Days	864.66	3.20	0.97	0.04	0.25
Season	866.37	4.91	1.00	0.05	0.25
Group	884.21	22.75	1.00	0.00	0.20
Intercept	885.23	23.77	1.00	0.01	0.20

(b)					
PCV model	AIC_c_	ΔAIC_c_	Cum. *w*AIC_c_	Marginal *R* ^2^	Conditional *R* ^2^
Days*Group	−302.26	0.00	1.00	0.35	0.53
Days	−255.92	46.34	1.00	0.05	0.48
Temp. prev.14	−248.85	53.41	1.00	0.05	0.42
Temp	−247.87	54.39	1.00	0.05	0.42
Temp. prev.3	−247.78	54.48	1.00	0.05	0.42
Temp. prev.7	−247.77	54.49	1.00	0.05	0.42
Season	−236.94	65.32	1.00	0.24	0.42
Group	−234.03	68.23	1.00	0.02	0.45
Intercept	−226.51	75.74	1.00	0.00	0.42

(c)					
TS Model	AIC_c_	ΔAIC_c_	Cum. *w*AIC_c_	Marginal *R* ^2^	Conditional *R* ^2^
Group	−378.13	0.00	0.97	0.46	0.75
Season	−370.70	7.44	1.00	0.01	0.75
Intercept	−364.45	13.68	1.00	0.00	0.75
Days	−363.21	14.92	1.00	0.00	0.75
Temp.prev.7	−362.60	15.53	1.00	0.00	0.75
Temp.prev.14	−362.58	15.55	1.00	0.00	0.75
Temp	−362.55	15.58	1.00	0.00	0.75
Temp.prev.3	−362.52	15.61	1.00	0.00	0.75

**Figure 4:**
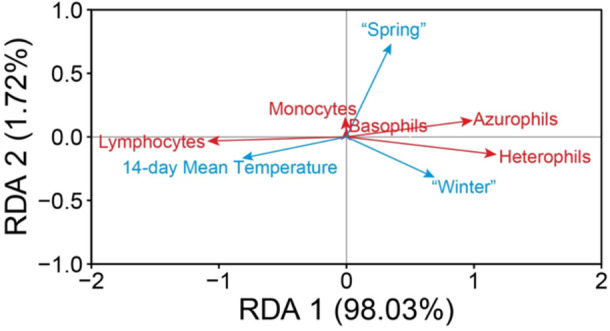
Redundancy analysis (RDA) of chi‐square‐transformed differential white blood cell counts with temperature and season in experimental animals. Spring and winter variables represent *sin* and *cos* of day of year converted to radians to represent the circular nature of these data. Each axis is labeled with the RDA axis it represents as well as the proportion of the total explained variance of each axis in the model. The direction and length of arrows represents each variable's loading along these axes. Variables pointing in the same or opposite angles are positively or negatively related, while orthogonal variables have no relationship to each other. The length of the arrow is correlated with the relative strength of each variable's effect.

Since total WBC was best explained by temperature, we used only the experimental group to conduct the RDA. The full model was significant (*p* < 0.001), while only the first axis (RDA1) was significant (*p* < 0.001), and the second axis (RDA2) was not significant (*p* = 0.104). Therefore, only the RDA1 axis provides any meaningful interpretation (Figure 4). Marginal tests showed that 14‐day mean temperature was the only significant independent variable (*p* < 0.001) while Spring and Winter were not significant predictors of WBC (*p* = 0.114 and *p* = 0.193, respectively). The RDA results show that increased 14‐day mean temperature was associated with higher lymphocytes and lower azurophils and heterophils (Figure 4). Monocytes and basophils were not significantly associated with any independent variable in this model.

## 4 Discussion

Hematologic assessment is one of the most common modalities to assess general health in wildlife, and often requires species‐specific reference ranges. Differences in hematologic variables within or between species are commonly attributed to demographic factors such as sex, age class, location, and season in temperate species. In vipers specifically, hematological variables have been shown to change seasonally (Troiano et al. 1997; LaGrange et al. 2014); and this has been historically attributed to intrinsic factors (i.e., physiologic cycles). However, in our study, WBC and PCV, two key components to the hematologic response, showed some level of temperature‐dependence that was not influenced by a temporal (seasonal) cycle in *C. viridis*. It is important to note that our experimental design manipulated photoperiod and feeding regime, in conjunction with temperature, as these variables naturally covary with temperature in temperate systems. Additionally, due to their ectothermic nature, snakes would be unable to properly digest food at low temperatures, potentially resulting in death. Therefore, the observed differences between control and experimental groups reflect the combined influence of correlated environmental variables. Nevertheless, the absence of a temporal cycle in WBC suggests that the environmental conditions associated with seasonal temperature variation, rather than an endogenous temporal cycle, are the primary drivers of WBC variation in this species.

The species‐specific nature of hematologic responses to environmental variation is underscored by contrasting results across reptilian taxa. Desert tortoises (*Gopherus agassizii*) exhibited completely temperature‐independent, seasonal variation in leukocytes with highest lymphocyte levels in the fall and winter (Sandmeier et al. 2016). Meanwhile, Blanding's turtles (*Emydoidea blandingii*) showed no significant difference in heterophils or lymphocytes when measured monthly (Mumm et al. 2019). In captive tortoise species, bactericidal ability against certain bacteria varied monthly but showed only limited relationships with thermal microenvironment which were both species‐ and pathogen‐specific (Hartzheim et al. 2023). In the grass snake (*Natrix natrix*), pronounced seasonal changes in red blood cell parameters were associated with breeding activity and hibernation, with winter lymphocytopenia and changes linked to both hormonal and temperature‐mediated processes (Wojtaszek 1992). In captive Louisiana pine snakes (*Pituophis ruthveni*), PCV and absolute heterophils differed between life stages, and WBC and lymphocytes were higher in adult males than females, highlighting the importance of accounting for intrinsic demographic factors alongside environmental variables (Giori et al. 2020). Similarly, plasma biochemical values in captive bearded dragons (*Pogona vitticeps*) varied by both sex and season, with differences in globulins, cholesterol, and calcium in females attributed to reproductive activity, and seasonal changes in uric acid linked to shifts in dietary intake and hydration associated with temperature variation (Tamukai et al. 2011). Collectively, these findings demonstrate that the relative importance of temperature, season, and intrinsic factors on hematologic parameters is likely taxon‐specific and warrants investigation across species of clinical or conservation concern.

The top model explaining variation in PCV showed an interactive effect between days since first blood collection and experimental group, suggesting some difference in resiliency to blood collection between these two groups. Snakes in the control group were kept at a constant, optimal temperature (25°C), while snakes in the experimental group were subjected to the full range of naturally‐occurring temperatures. Therefore, the snakes in the control group were potentially less physiologically stressed, allowing for better recovery from blood collection. Additionally, the experimental group experienced seasonal photoperiod changes and cessation of feeding when temperatures dropped below 20°C, both of which may have contributed to the observed decline in PCV. Photoperiod shifts and associated hormonal changes can influence erythropoietic activity in reptiles (Wojtaszek 1992), and food deprivation has been shown to decrease hematocrit in snakes, possibly through reduced erythropoiesis or changes in hydration status (Webb et al. 2017). Erythrocyte metrics are recognized as complex indicators in free‐living vertebrates, as both short‐term stress can affect PCV in opposing directions (Johnstone et al. 2017). Thus, the decline in PCV in our experimental group likely reflects the cumulative physiological impact of the full suite of seasonally cycling environmental conditions rather than any single variable. Free‐ranging Timber Rattlesnakes (*Crotalus horridus*) measured during the active season (April‐September) showed no significant variation in PCV (LaGrange et al. 2014), which may be indicative of the precision with which individual snakes can utilize behavioral thermoregulation to maintain optimal body temperatures. While PCV was tangentially connected to temperature through experimental group, WBC showed a negative relationship with temperature, where models including the mean temperature 14, 7, and 3 days prior to sampling were all equally supported. A study of captive boa constrictors (*Boa constrictor amarali)* found the opposite response in WBC, with higher values found in samples taken during the summer, although the authors did not report the temperature at the time of sampling (Machado et al. 2006). A study of captive tegu lizards (*Salvator merianae*) found no seasonal difference in WBC (Chamut and Arce 2018).

Analysis of individual leukocyte counts showed that temperature‐dependent variation of total WBC is driven by an increase in lymphocytes and azurophils and a decrease in heterophils with increasing temperatures. Using season as a proxy for temperature, higher lymphocytes were also observed in the summer in the Caspian Turtle (*Mauremys caspica*) (Muñoz and De la Fuente 2004). These results are important from a clinical standpoint as elevations in azurophils or lymphocytes could lead to an incorrect diagnosis of chronic bacterial, fungal, or viral disease. Additionally, in wild snakes, the temperature on the day or time of capture is often recorded but our results indicate it is not as relevant when assessing hematological data. Instead, our results show that recording the 14 day mean temperatures should be prioritized. More importantly, it is likely that temperature‐specific reference ranges are needed for each species. This helps captive management as hematologic findings of managed snakes can be compared across seasons if temperature remains constant.

Total solids, a measure of protein in the plasma, was influenced by treatment group independent of either temperature or season. This is likely the result of different feeding schedules between the treatment groups. Control snakes were fed every 2 weeks continuously throughout the study period, while feeding experimental group snakes ceased when their temperature was below 20°C. Plasma and acute phase proteins (APPs) are key players in the innate immune response, and increases (alpha, beta, and gamma globulins) or decreases (albumin) in concentrations indicate the presence of inflammation, infection, neoplasia, stress, or trauma (Cray 2012). Albumin, the largest component of total solids, is produced in the liver and associated with metabolism and therefore would naturally be higher in fed snakes. Globulins function as part of the immune system and their production requires a protein source, thus they would also be expected to be higher in fed snakes. Food deprivation in snakes has been shown to decrease circulating triglycerides and alter protein metabolism markers even over moderate time periods (Webb et al. 2017), consistent with the lower total solids observed in our experimental group, which experienced periods of fasting. These results support dietary intake as a critical variable to consider when interpreting plasma protein levels in reptiles, and that studies of free‐ranging animals in which feeding status is unknown should interpret total solids cautiously. Future investigations should pursue advanced proteomics or electrophoresis to determine the exact components of plasma protein and the influence of diet, season, temperature, and inflammation on each component.

## 5 Conclusion

Hematological research in understudied groups such as snakes is critical for increasing our understanding of their physiology and health and may ultimately lead to improvements in disease diagnosis and monitoring for reptiles. While temperature and season are correlated in many climates, the impacts of global climate change may result in unseasonable temperatures and shifts in season length. This study provides important baseline research for how those shifts may impact snake physiology. As global snake populations continue to decline (Reading et al. 2010), we will need to integrate data from all sub‐fields of biology to effectively achieve conservation and management goals.

## Data Availability

The data that support the findings of this study are available from the corresponding author upon reasonable request.
